# Exposición doméstica al ruido, problemas emocionales y trastorno de déficit de atención e hiperactividad en escolares de 9 años

**DOI:** 10.23938/ASSN.1079

**Published:** 2024-08-28

**Authors:** Irene Alfanjarín, Marisa Rebagliato, Marisa Estarlich, Amparo Cases, Ferran Ballester, Sabrina Llop, Maria-Jose Lopez-Espinosa, Llúcia González

**Affiliations:** 1 Universitat Jaume I Unitat Predepartamental de Medicina Castelló de la Plana Comunidad Valenciana España; 2 Instituto de Salud Carlos III Centro de Investigación Biomédica en Red de Epidemiología y Salud Pública (CIBERESP) Madrid España; 3 Universitat de València-Universitat Jaume I Unidad Mixta de Investigación en Epidemiología, Ambiente y Salud Fundación para el Fomento de la Investigación Sanitaria y Biomédica de la Comunidad Valenciana (FISABIO) Valencia España; 4 Universitat de València Facultat d’Infermeria i Podologia Valencia España; 5 Generalitat Valenciana Conselleria de Sanidad Centro de Salud Pública de Castellón Castelló de la Plana Comunidad Valenciana España; 6 Fundación para el Fomento de la Investigación Sanitaria y Biomédica de la Comunidad Valenciana (FISABIO) Valencia España

**Keywords:** Efectos del Ruido, Trastornos del Neurodesarrollo, Trastorno por Déficit de Atención con Hiperactividad, Niños, Trastornos del Sueño, Noise Effects, Neurodevelopmental Disorders, Attention Deficit Disorder with Hyperactivity, Child, Sleep Disorders

## Abstract

**Fundamento:**

Analizar la relación entre exposición al ruido en casa, problemas de sueño y riesgo de desarrollar problemas de neurodesarrollo en escolares de 9 años.

**Material y métodos:**

La exposición -frecuencia de molestia (baja, media, alta) por ruido de distintas fuentes percibida por 430 escolares de 9 años de la cohorte INMA-Valencia- fue informada por sus madres. El riesgo de desarrollar trastorno por déficit de atención e hiperactividad (TDAH), problemas internalizantes y problemas externalizantes se evaluó mediante el *Child Behavior Checklist*.

**Resultados:**

Las prevalencias de riesgo de problemas internalizantes (18%) y externalizantes (11,7%) fueron mayores que de TDAH (1,4%), y en niños mayores que en niñas. Las exposiciones más habituales y molestas se generaban en el propio hogar (50,8-55,3%) y por vecinos (24,5%). El riesgo de problemas de neurodesarrollo se asoció con problemas de sueño, especialmente para TDAH (16,1 vs 4%; p<0,001), sin diferencias por sexo. Los problemas de sueño fueron significativamente más frecuentes en escolares expuestos a ruido por otras actividades en el hogar o por vecinos. Altos niveles de exposición a tráfico en la calle y a vecinos predijeron riesgo de TDAH, mientras que a otros niños en casa predijo riesgo de problemas internalizantes y externalizantes; estos efectos no variaron al ajustar por problemas de sueño.

**Conclusiones:**

Altos niveles de molestia por ruido de distintas fuentes percibido en el hogar se relacionan de forma variable con el riesgo de distintos problemas en el neurodesarrollo de niños y niñas de 9 años, sin que los problemas de sueño afecten dicha relación.

## Abstract (en)

**Background:**

The purpose of this study was to assess the relationship between noise disturbance at home, sleep disturbance, and neurodevelopmental problems in 9-year-old children.

**Material and methods:**

Noise exposure (frequency) perceived by 430 9-year-old children from the *INMA* cohort in Valencia, Spain, were reported by their mothers. The risk of developing attention deficit hyperactivity disorder, as well as internalizing and externalizing problems, were assessed using the Child Behaviour Checklist.

**Results:**

The risk of internalizing (18%) and externalizing problems (11.7%) was higher compared to the risk of developing attention deficit hyperactivity disorder (1.4%) and were more prevalent in boys than in girls. The most common and bothersome noise exposures were generated at home (50.8-55.3%) and by neighbours (24.5%). The risk of neurodevelopmental problems was associated with sleep disturbances, particularly in relation with attention deficit hyperactivity disorder (16.1 vs 4%; p<0.001), with no differences observed between sex. Sleep disturbances were significantly more common in children exposed to noise from household or neighbours. High levels of noise exposure from street traffic and neighbours were linked to an increased risk of attention deficit hyperactivity disorder, while noise from other children at home was associated with a higher risk of internalizing and externalizing problems. These effects remained consistent even after adjusting for sleep disturbances.

**Conclusions:**

High levels of noise annoyance from various sources perceived at home are differently associated with the risks of different neurodevelopmental problems in 9-year-old boys and girls, with sleep disturbances not influencing this relationship.

## INTRODUCCIÓN

Según la Agencia Europea de Mediambiente (EEA, *European Environment Agency*)^1^, 113 millones de personas en la Unión Europea, y aproximadamente el 29% de la población española, estaban en 2017 expuestas al ruido del tráfico con niveles superiores a los recomendados por la Organización Mundial de la Salud (OMS) (máximo 55 decibelios de día y 50 de noche)^2^. El 29% de la población española está expuesta a niveles de ruido por tráfico que superan ese límite^1^. El ruido del transporte o la industria tienen un impacto menor en la población general, pero son una fuente importante de contaminación acústica a nivel local^2^. Dicha contaminación supone, por tanto, un problema a causa del cual se pierden cada año en Europa Occidental entre 1 y 1,6 millones de años de vida, calculados según años de vida ajustados por discapacidad^1^.

Existe evidencia cada vez más consistente de las consecuencias sobre la salud por exposición al ruido ambiental, el cual se ha relacionado con ansiedad, depresión, y enfermedades neurodegenerativas y cardiovasculares en personas adultas^3^^,^^4^. Tanto en la etapa adulta como en la infantil se asume que la exposición al ruido estimula repetidamente los sistemas endocrino y nervioso autónomo, desencadenando un estado de hiperexcitabilidad fisiológica^1^^) (^^5^^-^^11^. La etapa infantil presenta ciertas vulnerabilidades porque durante ella se carece de habilidades de anticipación y afrontamiento ante factores estresantes^4^^,^^11^ y coincide con el periodo de máxima plasticidad neuronal, por lo que cualquier efecto puede modular el neurodesarrollo^4^^,^^8^^,^^9^^,^^11^.

El impacto del ruido en la salud y desarrollo infantil está menos estudiado, predominando las investigaciones centradas en la exposición al ruido del tráfico en el entorno escolar respecto a otras fuentes o en el hogar, con resultados heterogéneos^12^^-^^14^. Aunque los niveles de ruido del tráfico en la escuela y en los hogares pueden estar correlacionados, se han observado más asociaciones significativas en el hogar que en el colegio entre la exposición al ruido con hiperactividad y con problemas de falta de atención^15^, porque durante la niñez se pasa más tiempo en casa que en la escuela y, además, la exposición nocturna al ruido del tráfico se ha asociado con alteraciones del sueño, tanto en calidad como en cantidad^15^.

Existe evidencia de que el sueño es una tercera variable que puede mediar en la asociación entre el ruido y el neurodesarrollo infantil^16^^,^^17^ ya que las alteraciones del sueño debidas al ruido nocturno causarían hipotimia, cansancio y peor desempeño de tareas escolar^16^.

Entre los problemas neuropsicológicos asociados al ruido durante la infancia se encuentra el trastorno por déficit de atención e hiperactividad (TDAH)^6^, con una prevalencia de 3,4-5,3% y caracterizado por la inatención, hiperactividad o impulsividad manifestadas en dos o más ambientes y durante seis o más meses^6^. Otros problemas como los internalizantes (experimentados como una angustia interna) y externalizantes (caracterizados por la irritabilidad, agresividad y ruptura de normas) podrían estar relacionados con la exposición al ruido, aunque los resultados no son concluyentes^3^^,^^15^^,^^16^; su prevalencia en la infancia es de 7,9-11,2%^7^ y 8,7-14,7%^3^^,^^7^, respectivamente.

El presente estudio forma parte del Proyecto INMA (INfancia y Medio Ambiente)^18^^,^^19^ cuyo objetivo principal es investigar la potencial influencia de los contaminantes ambientales en el embarazo y durante los primeros años de la vida, así como evaluar sus efectos sobre el crecimiento, salud y desarrollo infantil.

Teniendo en cuenta el incremento de la contaminación acústica, la prevalencia de los trastornos del neurodesarrollo y los costes sociales y económicos que de ellos se derivan, es interesante alcanzar una comprensión más profunda del potencial papel de la percepción del ruido ambiental como factor de riesgo, para así poder actuar en la prevención de estos trastornos y alteraciones.

Por todo lo expuesto, el presente estudio tiene como objetivos: 1) describir el grado de molestia de las diferentes fuentes de ruido presentes en los hogares de familias de la cohorte INMA-Valencia, 2) describir el riesgo de problemas internalizantes, externalizantes y TDAH en población infantil de 9 años de la cohorte INMA-Valencia, y 3) estudiar la relación entre el grado de molestia acústica en el domicilio y el riesgo de presentar TDAH y problemas internalizantes y externalizantes en dicha población, considerando otros factores potencialmente influyentes, como los problemas de sueño.

## MATERIAL Y MÉTODOS

Estudio transversal enmarcado en el Proyecto INMA (Infancia y Medio Ambiente), estudio multicéntrico de cohortes localizadas en diferentes áreas de la geografía española: Asturias, Granada, Gipuzkoa, Menorca, Ribera de l’Ebre, Sabadell y Valencia. Cada cohorte tiene diferentes periodos y tiempos de reclutamiento. El seguimiento ha experimentado ligeras variaciones entre cohortes, pero todas han llevado a cabo los seguimientos principales durante el embarazo, al parto, al año y a los cuatro o cinco años, siete años y nueve años^18^^,^^19^.

Este estudio utiliza información de la cohorte de la provincia de Valencia (España), iniciada en 2003 y constituida por población infantil de zonas urbanas, metropolitanas, semiurbanas y rurales correspondientes a los antiguos departamentos de salud 6 y 7. El reclutamiento de mujeres embarazadas se llevó a cabo en el Hospital La Fe (Valencia, España), durante la visita prenatal del primer trimestre para la realización del cribado poblacional de malformaciones congénitas.

Se ofreció participar en el proyecto a todas las embarazadas que cumplieran los siguientes criterios de inclusión: tener al menos 16 años, embarazo no gemelar ni múltiple, estar entre la semana 10 y la 13 de gestación, no haber seguido procedimientos de fertilización asistida, y tener intención de dar a luz en el Hospital La Fe. De 855 mujeres embarazadas incluidas al inicio, tras los diferentes seguimientos se produjeron 31 abortos, 4 muertes fetales, 5 fallecimientos, 226 abandonos, 100 pérdidas de seguimiento y 58 pérdidas por otras causas, dejando 431 niños y niñas participantes a los 9 años.

El estudio fue aprobado en su inicio por el Comité de Ética del Hospital La Fe de Valencia, donde se llevó a cabo en reclutamiento de las mujeres embarazadas. La visita de seguimiento de los 9 años fue aprobada por el Comité de Ética de la Fundación para el Fomento de la Investigación Sanitaria y Biomédica de la Comunitat Valenciana, y respeta los principios de la Declaración de Helsinki. Los progenitores de los participantes seleccionados, tras recibir información del estudio por escrito y de forma oral, firmaron un consentimiento informado.

### Variable de exposición

El grado de molestia que siente el niño o niña a causa del ruido se evaluó mediante un cuestionario *ad hoc* de tipo Likert respondido por las madres (material suplementario, S1). Se consideraron ocho fuentes de ruido: 1) presencia de otros niños en casa, 2) realización de otras actividades en casa (televisión, radio, presencia de adultos), 3) gente en la calle, 4) tráfico en la calle, 5) vecinos, 6) locales de ocio (bares, pubs, discotecas), 7) talleres o industrias, 8) obras (públicas en la calle o privadas en hogar/inmueble). De cada fuente se detalló si era habitual percibirlas y su frecuencia: a= muy a menudo, b= bastante, c= a veces, d= casi nunca, e= nunca, agrupadas en exposición alta (a/b), media (c) y baja (d/e).

### Variables de efecto

Los tres resultados de salud estudiados fueron el TDAH y los problemas internalizantes y externalizantes, cuya presencia fue evaluada mediante el cuestionario *Child Behavior Checklist* (CBCL), aplicable de 6 a 18 años de edad^20^^,^^21^. Este cuestionario pertenece al sistema de evaluación multiaxial ASEBA (*Achenbach System of Empirically Based Assessment*) para valorar psicopatología^20^^,^^21^ y es respondido por los progenitores. Está constituido por dos partes diferenciadas; la primera trata sobre las actividades del niño y la segunda valora su comportamiento. En esta investigación solo se utilizó la segunda, formada por 113 ítems que describen la conducta del niño en los últimos seis meses con tres opciones de respuesta (0= nunca o casi nunca, 1= a veces, 2= muy a menudo o bastante a menudo). Los ítems se agrupan en ocho escalas sindrómicas: 1) ansiedad/depresión, 2) retraimiento/depresión, 3) quejas somáticas, 4) problemas sociales, 5) problemas de pensamiento, 6) problemas de atención, 7) conducta disruptiva y 8) conducta agresiva. Sin embargo, los creadores del CBCL, también permiten la recombinación de los ítems en las siguientes escalas DSM, equiparables a los criterios del *Diagnostic and Statistical Manual of Mental Disorders*^20^^-^^23^: a) problemas afectivos, b) problemas de ansiedad, c) problemas somáticos, d) TDAH, e) trastorno de oposición y f) problemas de conducta. Tanto para las escalas sindrómicas como para las escalas DSM se trasladaron las puntuaciones crudas obtenidas a un baremo para obtener las puntuaciones típicas y los percentiles. Se ha utilizado la escala d (DSM) de TDAH y las escalas de problemas internalizantes y externalizantes, que son agrupaciones de las escalas sindrómicas. Las tres tienen un rango de 0 a 100 y se utilizaron los puntos de corte utilizados por el manual, que toman como referencia los percentiles:

- TDAH (escala d DSM): según el percentil alcanzado, se clasificó a los participantes en riesgo bajo (P0-P92), medio (P93-P97) o alto (P≥98)^24^.
- El riesgo de problemas internalizantes se calculó considerando las escalas sindrómicas 1 (ansiedad/depresión), 2 (retraimiento/depresión) y 3 (quejas somáticas), y el riesgo de problemas externalizantes considerando las escalas sindrómicas 7 (conducta disruptiva) y 8 (conducta agresiva). Se clasificó a los participantes en riesgo bajo (P0-P83), medio (P84-P90) o alto (P≥91)^24^. Estas tres categorías se recodificaron en la variable binaria riesgo: bajo (P0-P83) y medio-alto (P≥84).

### Variables de control

- Sociodemográficas de los progenitores:
  - edad (en años): <25, 25-29, 30-34, ≥35;
  - nivel de estudios: primarios/ESO, secundarios, universitarios;
  - lugar de residencia: urbano, metropolitano, semiurbano, rural, fuera del área;
  - clase social basada en la ocupación codificada mediante la adaptación del sistema nacional de ocupaciones (CNO-94)^25^: alta: I/II, media: III, baja: IV/V;
  - situación laboral: empleada/baja médica, desempleada, otros;
  - país de origen: España, otro (extranjero);
  - percepción subjetiva de la economía familiar: cómoda, bien, tirando, difícil, muy difícil, no sabe/no contesta.
- Psicológicas de los progenitores: la inteligencia y la salud mental se evaluaron con dos cuestionarios que cuentan con buenas propiedades psicométricas y son utilizados a menudo en estudios epidemiológicos:
  - inteligencia: medida mediante el test de semejanzas de la *Wechsler Adult Intelligence Scale III* (WAIS-III)^26^, test que valora la comprensión y formación verbal de conceptos, el razonamiento abstracto y lógico, el pensamiento asociativo, la habilidad de separar detalles esenciales y no esenciales y la memoria; puntúa de 0 a 35;
  - salud mental: evaluada mediante el *Symptom Checklist-90-Revised* (SCL-90-R)^27^ que valora la presencia de 90 síntomas, estableciendo su intensidad en una escala que va desde la ausencia total hasta la máxima intensidad (rango de puntuaciones típicas: 0-100; punto de corte para riesgo: ≥65).
- Características del entorno familiar:
  - Estilo de vida de la madre durante el embarazo: consumo de tabaco (sí, no) y consumo de alcohol (sí, no).
  - otros convivientes del niño menores de 16 años (número);
  - el padre convive con la madre y descendientes (sí, no).
-Características del niño:
  - sexo: femenino, masculino;
  - edad (años),
  - bajo peso al nacer (<2.500 g): sí, no;
  - pretérmino (<semana 37): sí, no;
  · pequeño para la edad gestacional: sí, no;
  - semanas de lactancia: 0, 1-16, 17-24, ≥25;
  - problemas de sueño: se calculó una puntuación (rango 0-5) a partir de los ítems 76 (dormir menos), 77 (dormir más), 92 (somniloquia o sonambulismo), y 100 (no dormir bien) del CBCL, que en estudios anteriores presentaba correlación con otras escalas validadas específicas para evaluar los trastornos de sueño en la infancia^28^. La puntuación se dicotomizó en 0= sin problemas y 1-5= con problemas.

Las variables categóricas se describieron mediante porcentajes y se compararon con la prueba Chi-cuadrado (χ2), mientras las continuas se describieron con medianas y rangos intercuartílicos (RIC) y se compararon entre varios grupos con Kruskal-Wallis; la edad se describió con media y desviación estándar (DE).

La relación entre la molestia por ruido y el riesgo binario de trastornos (TDAH, problemas internalizantes y problemas externalizantes) se analizó mediante regresión logística múltiple. Se incluyeron en cada modelo inicial todas las variables asociadas al riesgo de estos trastornos en el análisis univariante (p<0,05) y se aplicó el procedimiento por pasos hacia atrás (*step backwards*) con criterio de salida p >0,20 según el test de la razón de verosimilitud (LR). A continuación, se incluyeron aquellas variables de ruido presentes en al menos el 10% de los trastornos y con p <0,10 en el análisis univariante. Ante el posible efecto mediador de los problemas de sueño en la relación ruido-trastornos, esta variable no se incluyó inicialmente en los modelos principales sino que, primeramente, se valoró si actuaba como una variable de confusión (relacionada tanto con las distintas fuentes de ruido como con el riesgo de trastornos) y, en segundo lugar, como análisis de sensibilidad, se incluyó en los modelos multivariantes para ver cómo afectaba a los estimadores de las diferentes variables de ruido. Los modelos finales muestran la *odds ratio* (OR) y su intervalo de confianza al 95% (IC95%) como estimador de cada variable ajustado por el resto de variables.

El análisis se realizó con *SPSS Statistics*, versión 27. Las figuras fueron creadas con *Datawrapper*, R y *RStudio* versiones 4.1.3 y 2022.02.3+492, respectivamente, y con el paquete ggplot2.

## RESULTADOS

La muestra inicial estaba compuesta por 431 unidades familiares con hijos de 9 años. Una madre que asistió a la visita de seguimiento pero dejó el cuestionario incompleto, por lo que la muestra con datos completos sobre la variable de exposición contó con 430 participantes. Además, en tres casos los progenitores no cumplimentaron el cuestionario para evaluar los problemas internalizantes, externalizantes y el TDAH (CBCL), por lo que solo se pudo evaluar en 427 participantes.

En la Tabla 1 se presentan las variables estudiadas en la muestra. La mayoría de madres y padres participantes pertenecía a la clase social más baja, tenía entre 30 y 34 años, era de origen español, y estaban empleados. Predominó el nivel de estudios secundarios en madres y primarios en padres. Solo un 15% percibía su economía familiar como difícil o muy difícil, y más del 70% indicaron vivir en el área metropolitana o semiurbana. Menos del 8%, tanto de madres como de padres, tuvo problemas de salud mental, y más del 17% de mujeres consumieron tabaco o alcohol durante el embarazo. Respecto a los niños participantes (49,4% niñas), su edad promedio era 9,13 años (DE= 0,24; RIC= 8,94-9,26) y la mayoría convivían con su padre y con algún hermano o hermana menor de 16 años. Solo un 5% habían nacido pretérmino o con bajo peso, y casi la mitad lactó durante más de 24 semanas. El 41% presentaban problemas de sueño.

**Tabla 1:** Características de la muestra de la cohorte INMA-Valencia en estudio

	Madre n(%)	Padre n(%)		n (%)
Características de progenitores desagregadas por sexo			Características de la familia y del niño participante	
*Características socioeconómicas*			*Características socioeconómicas de la familia*	
Clase social	n=430	n=429	Clase social familiar, n=430	
I+II (alta)	82 (19,0)	71 (16,6)	I+II (alta)	118 (27,4)
III (media)	117 (27,3)	81 (18,9)	III (media)	116 (27,0)
IV+V (baja)	231 (53,7)	277 (64,6)	IV+V (baja)	196 (45,6)
Edad (en años)	n=430	n=430	Lugar de residencia, n=417	
< 25	30 (7,0)	27 (6,3)	Urbano	36 (8,6)
25-29	144 (33,5)	125 (29,1)	Metropolitano	174 (41,6)
30-34	185 (43,0)	173 (40,2)	Semiurbano	149 (35,7)
≥35	71 (16,5)	105 (24,4)	Rural	29 (7,0)
País de origen	n=430	n=430	Fuera del área	29 (7,0)
España	405 (94,2)	387 (90,0)	Percepción de la economía familiar, n=428	
Extranjero	25 (5,8)	43 (10,0)	Cómoda	56 (13,1)
Estudios	n=430	n=428	Bien	149 (34,8)
Primarios/ESO	116 (27,0)	184 (43,0)	Va tirando	155 (36,2)
Secundarios	189 (44,0)	169 (39,5)	Difícil	41 (9,6)
Universitarios	125 (29,0)	75 (17,5)	Muy difícil	23 (5,4)
Situación laboral	n=429	n=427	Ns/Nc	4 (0,9)
Empleada / baja	292 (68,1)	348 (81,5)	*Características del entorno familiar, n=430*	
Otros	49 (11,4)	11 (2,6)	El padre vive con madre e hijos (sí)	355 (82,6)
Desempleada	88 (20,5)	68 (15,9)	Convivientes del niño <16 años	
*Características psicológicas*			0	151 (35,1)
Salud mental (SCL-90-R)	n=413	n=319	1	246 (57,2)
Con riesgo	27 (6,5)	25 (7,2)	>1	33 (7,7)
Inteligencia (WAIS-III)	n=415	n=126	*Características del niño, n= 429*	
mediana (RIC)	9,76 (8,29-11,97)	9,13 (7,35-11,52)	Sexo (femenino)	
*Estilo de vida materno durante el embarazo, n=430*			Pretérmino (sí)	23 (5,4)
			Pequeño para la edad gestacional (sí)	52 (12,1)
Tabaco			Bajo peso al nacer (sí)	24 (5,6)
s.12 (sí)	91 (21,2)		Semanas de lactancia, n= 430	
s.32 (sí)	84 (19,5)		0	68 (15,8)
Alcohol			1-16	96 (22,3)
s.12 (sí)	75 (17,6)		17-24	68 (15,8)
s.32 (sí)	76 (17,7)		≥25	198 (46,0)
			Problemas de sueño (sí), n=423	174 (41,1)

RIC: rango intercuartílico; SCL-90-R: *Symptom Checklist-90-Revised*; WAIS: *Wechsler Adult Intelligence Scale*.

Los trastornos del neurodesarrollo más frecuentes fueron los problemas internalizantes, seguidos de los problemas externalizantes y del TDAH. Las prevalencias para riesgo alto de TDAH, y externalizantes fueron 1,4%, 18,0%, y 11,7%, respectivamente (Tabla 2). Cuando se consideraron las tres categorías de riesgo, los niños presentaron significativamente mayores prevalencias de riesgo que las niñas, especialmente de TDAH (2,3 vs. 0,5%) pero también de problemas internalizantes.

**Tabla 2:** Prevalencia de trastornos de neurodesarrollo en la muestra, global y por sexo

Riesgo de	Global	Niños	Niñas	p
	n (%)	n (%)	n (%)	(χ2)
TDAH				0,048
Bajo	390 (91,1)	201 (92,6)	189 (89,6)	
Medio	32 (7,5)	11 (5,1)	21 (10,0)	
Alto	6 (1,4)	5 (2,3)	1 (0,5)	
Problemas internalizantes				0,004
Bajo	303 (70,8)	138 (63,3)	165 (78,2)	
Medio	48 (11,2)	31 (14,3)	17 (8,1)	
Alto	77 (18,0)	48 (22,1)	29 (13,7)	
Problemas externalizantes				0,152
Bajo	337 (78,7)	163 (75,1)	174 (82,5)	
Medio	41 (9,6)	23 (10,6	18 (8,5)	
Alto	50 (11,7)	31 (14,3)	19 (9,0)	

TDAH: Trastorno por Déficit de Atención e Hiperactividad.

Las fuentes de ruido más habituales referidas por las madres se generaban en la mitad de los casos en el propio domicilio (55,3% por presencia de otros niños y 50,8% por realización de actividades) y en el 24,5% en el inmueble (vecinos). Estas eran las mismas fuentes que las madres identificaron como aquellas que podían causar un alto grado de molestia a sus hijos con mayor frecuencia (14,2%, 7,9% y 3,5%, respectivamente) (Tabla 3).

**Tabla 3:** Presencia de fuentes de ruido y grado de molestia para los hijos percibido por las madres

Fuentes de ruido	Presencia habitual n (%)	Grado de molestia*		
		Alto n (%)	Medio n (%)	Bajo n (%)
Otros niños en la casa	238 (55,3)	61 (14,2)	99 (23,0)	270 (62,8)
Otras actividades en la casa	218 (50,8)	34 (7,9)	94 (21,9)	302 (70,2)
Gente en la calle	69 (16,1)	5 (1,2)	30 (7,0)	395 (91,9)
Tráfico en la calle	83 (19,3)	7 (1,6)	15 (3,5)	408 (94,9)
Vecinos	105 (24,5)	15 (3,5)	36 (8,4)	379 (88,1)
Bares, pubs, discotecas	30 (7,0)	6 (1,4)	12 (2,8)	412 (95,8)
Talleres, industrias	14 (3,3)	2 (0,5)	1 (0,2)	426 (99,3)
Obras (públicas o privadas)	10 (2,3)	1 (0,2)	12 (2,8)	417 (97,0)

*: Alto: bastante o muy a menudo, Medio: a veces, Bajo: nunca o casi nunca.

Los problemas de neurodesarrollo se asociaron a los problemas de sueño, tanto globalmente como en niños y en niñas, de forma significativa excepto para los problemas externalizantes en niños. La frecuencia de riesgo medio/alto de TDAH se cuatriplicó en los participantes con problemas de sueño respecto a los que no los tenían (16,1 vs 4%) y la de problemas internalizantes se duplicó (19,8% vs. 42,0%), mientras que la de externalizantes se multiplicó por 1,6 (Tabla 4A). No hubo diferencias por sexo, si bien el aumento de problemas internalizantes y externalizantes fue más acusado en niñas que en niños.

Los problemas de sueño se presentaban con mayor frecuencia en aquellos participantes con percepción de molestia alta en cada una de las fuentes de ruido, aunque la diferencia solo fue estadísticamente significativa para el ruido causado por otras actividades en la casa o por los vecinos (Tabla 4B). El ruido en casa ocasionado por la presencia de otros niños y por actividades se asoció con el riesgo de los tres problemas de neurodesarrollo; el TDAH se asoció también al ruido del tráfico y de vecinos, y los problemas internalizantes al ruido de la gente en la calle. El ruido ocasionado por otras actividades en la casa y por vecinos se vinculó a los problemas de sueño (Tabla 4B).

**Tabla 4A:** Asociación entre el riesgo (medio/alto frente a bajo) de desarrollar problemas de neurodesarrollo y la presencia de problemas de sueño

	Problemas de sueño	TDAH			Problemas internalizantes			Problemas externalizantes		
		n	(%)	p (χ2)	n	(%)	p (χ2)	n	(%)	p (χ2)
Global	No	10	(4,0)	<0,001	49	(19,8)	<0,001	42	(16,9)	0,009
	Sí	28	(16,1)		73	(42,0)		48	(27,6)	
Niños	No	4	(3,1)	0,003	36	(28,3)	0,004	27	(21,3)	0,137
	Sí	12	(14,0)		41	(47,7)		26	(30,2)	
Niñas	No	6	(4,9)	0,002	13	(10,7)	<0,001	15	(12,4)	0,017
	Sí	16	(18,2)		32	(36,4)		22	(25,0)	

TDAH: Trastorno por Déficit de Atención e Hiperactividad.

**Tabla 4B:** Asociación del grado de molestia ocasionada por cada una de las fuentes de ruido con la frecuencia de problemas de sueño y del neurodesarrollo

	Problemas de sueño		TDAH		Problemas internalizantes		Problemas externalizantes	
Grado de exposición al ruido	n (%)	p(χ2)	n (%)	p(χ2)	n (%)	p(χ2)	n (%)	p(χ2)
Niños y niñas en la casa		0,423		0,001		<0,001		<0,001
Media-baja	146 (40,4)		26 (7,1)		92 (25,1)		64 (17,5)	
Alta	28 (45,9)		12 (19,7)		33 (54,1)		27 (44,3)	
Actividades en la casa		0,030		0,002		<0,001		0,012
Media-baja	154 (39,7)		30 (7,6)		103 (26,2)		78 (19,8)	
Alta	20 (58,8)		8 (23,5)		22 (64,7)		13 (38,2)	
Gente en la calle		0,391		0,381		0,012		0,034
Media-baja	171 (41,0)		37 (8,8)		121 (28,7)		88 (20,9)	
Alta	3 (60,0)		1 (20,0)		4 (80,0)		3 (60,0)	
Tráfico en la calle		0,102		0,001		0,967		0,160
Media-baja	169 (40,7)		35 (8,3)		123 (29,3)		88 (21,0)	
Alta	5 (71,4)		3 (42,9)		2 (28,6)		3 (42,9)	
Vecinos		0,010		<0,001		0,725		0,606
Media-baja	163 (40,1)		32 (7,8)		120 (29,1)		87 (21,1)	
Alta	11 (73,3)		6 (40,0)		5 (33,3)		4 (26,7)	

TDAH: Trastorno por Déficit de Atención e Hiperactividad.

La Tabla 5 muestra las covariables que se asociaron significativamente con el riesgo de presentar problemas de neurodesarrollo (en la Tabla S2 del material suplementario se muestran el resto de variables consideradas que no alcanzaron significación estadística). Se observó una relación entre mayor riesgo (medio/alto) de los problemas de neurodesarrollo estudiados y menor clase social, estudios maternos y paternos más bajos y tener padres desempleados. Se observó un gradiente donde los riesgos aumentaban con la mala percepción de la economía familiar, y correlacionaban positivamente con la mala salud mental de la madre. Para problemas internalizantes, se observó más riesgo en padres con mala salud mental y con menor puntuación en el test de inteligencia. El sexo masculino se asoció a más problemas internalizantes, y no haber tenido bajo peso al nacer, a los externalizantes.

**Tabla 5:** Covariables relacionadas con riesgo medio/alto frente a bajo de TDAH, problemas internalizantes y externalizantes

	TDAH	p(χ2)	Problemas internalizantes	p(χ2)	Problemas externalizantes	p(χ2)
Características socioeconómicas						
Clase social madre						
I+II (alta)	2,5	0,011	16,0	0,011	16,0	0,178
III (media)	6,1		29,6		18,3	
IV+V (baja)	12,5		33,6		24,6	
Clase social padre						
I+II (alta)	2,8	0,004	18,3	0,032	9,9	0,027
III (media)	2,5		24,7		19,8	
IV+V (baja)	12,0		33,1		24,4	
Clase social conjunta						
I+II (alta)	3,4	0,004	18,8	0,011	16,2	0,224
III (media)	6,1		30,4		20,9	
IV+V (baja)	13,8		34,7		24,5	
Nivel educativo madre						
Primarios/ESO	20,5	<0,001	41,0	<0,001	30,8	<0,001
Secundarios	5,3		29,4		23,0	
Universitarios	3,2		17,7		9,7	
Nivel educativo padre						
Primarios/ESO	12,6	0,012	33,9	0,003	29,0	<0,001
Secundarios	7,7		31,0		19,0	
Universitarios	1,3		13,3		6,7	
Situación laboral del padre						
Empleado + baja	6,6	0,003	27,1	0,153	18,4	0,034
Otros	10,0		30,0		40,0	
Desempleado	19,4		38,8		29,9	
Percepción subjetiva de la economía familiar						
Cómodamente	5,4	0,003	26,8	0,027	16,1	0,014
Va bien	4,1		23,0		14,2	
Va tirando	10,5		30,7		23,5	
Bastante difícil	17,1		34,1		34,1	
Muy difícil	26,1		56,6		39,1	
Ns/Nc	0,0		50,0		25,0	
Características psicológicas de los progenitores						
Salud mental madre (SCL-90-R)						
Sin riesgo	7,5	<0,001	27,0	<0,001	19,0	<0,001
Con riesgo	33,3		70,4		59,3	
Salud mental padre (SCL-90-R)						
Sin riesgo	8,6	0,617	27,2	0,004	18,8	0,596
Con riesgo	11,5		53,8		23,1	
Inteligencia del padre (WAIS-III)* (n=126)						
mediana (RIC)	7,05 (2,14-11,96)	0,086	8,54 (6,16-11,22)	0,032	8,84 (6,00-11,07)	0,1
Características del niño						
Sexo						
Femenino	10,4	0,267	21,8	0,001	17,5	0,063
Masculino	7,4		36,4		24,9	
Bajo peso al nacer			
No	8,9	0,975	30,1	0,08	22,2	0,042
Sí	8,7		13,0		4,3	

TDAH: Trastorno por Déficit de Atención e Hiperactividad; SCL-90-R: *Symptom Checklist-90-Revised*; WAIS: Wechsler Adult Intelligence Scale; Ns/Nc: no sabe/no contesta; *: comparadas con Kruskall-Wallis.

Los modelos finales de regresión logística múltiple para la probabilidad de riesgo medio/alto de desarrollar cada problema de neurodesarrollo están ajustados por el nivel de exposición alto (frente a molestia baja/media) de las fuentes de ruido y por otras covariables (Tabla 6). Respecto del análisis univariante mostrado en la Tabla 4B, la alta exposición a las fuentes de ruido que retuvieron la significación estadística en el modelo ajustado para TDH fueron el tráfico en la calle y los vecinos, para los problemas internalizantes fueron otros niños en la casa y actividades en la casa, mientras que solo el ruido producido por otros niños en la casa fue predictor independiente para problemas externalizantes. Al incluir los problemas de sueño en el modelo final se observó que los estimadores se mantuvieron en rangos equivalentes al análisis principal, incrementándose ligeramente en el caso de niños en la casa y gente en la calle, y reduciéndose para el resto de las fuentes de ruido.

La asociación individual y ajustada de la exposición alta a cada fuente de ruido con los problemas de neurodesarrollo se muestra en las figuras S3 y S4 del material suplementario.

**Tabla 6:** Asociación entre el alto nivel de exposición a distintas fuentes de ruido y el riesgo medio/alto de desarrollar trastornos del neurodesarrollo

Grado de molestia alto por ruido	TDAH		Problemas internalizantes		Problemas externalizantes	
	OR (IC95%)	p	OR (IC95%)	p	OR (IC95%)	p
*Modelos principales*						
Niños en la casa	2,37 (0.90-6,26)	0,081	4,21 (2,06-8,63)	<0,001	3,85 (1,95-7,61)	<0,001
Actividades en la casa	3,16 (0,99-10,06)	0,052	3,64 (1,53-8,66)	0,004	1,45 (0,58-3,64)	0,432
Gente en la calle			2,69 (0,24-30,72)	0,426	4,33 (0,46-40,50)	0,199
Tráfico en la calle	13,12 (1,82-94,55)	0,011	-		-	
Vecinos	5,04 (1,12-22,61)	0,035	-		-	
*Modelos añadiendo problemas de sueño*						
Niños en la casa	2,6 (0,93-7,26)	0,068	4,7 (2,25-9,83)	<0,001	3,98 (2,00-7,92)	<0,001
Actividades en la casa	2,33 (0,69-7,85)	0,171	3,28 (1,34-8,04)	0,009	1,32 (0,52-3,38)	0,564
Gente en la calle			2,68 (0,24-30,43)	0,426	4,47 (0,46-43,47)	0,197
Tráfico en la calle	10,39 (1,52-71,26)	0,017	-		-	
Vecinos	4,64 (1,10-19,59)	0,037	-		-	
Variables de ajuste	Situación laboral padre, economía familiar,tipo de familia		Nivel de estudios padre, bajo peso al nacer, nº convivientes <16		Nivel de estudios padre, situación laboral madre, clase social familiar, economía familiar, bajo peso al nacer	

TDAH: Trastorno por Déficit de Atención e Hiperactividad; OR: *odds ratio*; IC: intervalo de confianza.

## DISCUSIÓN

La prevalencias de riesgo alto de TDAH en los participantes de este estudio (1,4%) fue inferior a la estimada en la infancia y adolescencia por un metaanálisis^6^ (3,40-5,29%); la diferencia puede deberse, además de a la diferencia de rango de edad, a que utilizamos datos de un cuestionario mientras que el metaanálisis también incluyó diagnósticos clínicos. Si agregamos las categorías de riesgo medio y alto, la prevalencia (9,1%) se acerca más a la estimada por la OMS para esta edad (5-8%)^3^.

En cuanto a la prevalencia para problemas internalizantes y externalizantes, un estudio europeo realizado en población de 6-12 años^29^, reportó porcentajes de problemas internalizantes y externalizantes del 18,4%, y 7,8%, muy similares a los obtenidos en este estudio. Otro trabajo europeo^30^ realizado en niños y niñas de 6-11 años obtuvo porcentajes inferiores (3,8% en internalizantes y 8,4% en externalizantes); las diferencias podrían deberse a que las prevalencias se determinaron con un instrumento distinto al empleado en nuestro estudio y a que predominó la participación de población de Europa del Este respecto de la Occidental, lo que podría suponer una diferencia en cuanto a poblaciones estudiadas.

En este trabajo se observó que los hogares ruidosos tenían mayor riesgo de presentar estos trastornos del neurodesarrollo. El ruido de tráfico se relacionó con un aumento de riesgo de desarrollar TDAH, coincidiendo con la mayoría de los estudios de un metaanálisis de 2019^12^, aunque se suelen centrar en el entorno escolar. No obstante, se ha comprobado que esta asociación es más significativa cuando la exposición es a nivel domiciliario^12^, debido a que implica un mayor tiempo de exposición y a que la exposición nocturna puede conllevar alteraciones en el sueño^12^^,^^16^^,^^17^.

Las molestias por el ruido generado por la presencia de otros niños en la casa mostraron una relación fuerte con los problemas internalizantes y externalizantes, y las producidas por el ruido causado por otras actividades en la casa con los problemas internalizantes. Parte del efecto atribuido al ruido causado por otros niños en el domicilio podría relacionarse con otros factores, y al ruido producido por actividades en la casa podrían contribuir los hermanos del participantea^28^^,^^31^. El número de niños o de actividades más ruidosas en la casa podría ser un indicador indirecto de un bajo nivel socioeconómico, y son precisamente estos los hogares con mayor número de hijos^32^^,^^33^. Estos hogares se ubican, con frecuencia, en domicilios peor aislados acústicamente y situados en zonas más ruidosas^32^^-^^34^, aunque en este estudio no observamos relación significativa entre el lugar de residencia y el riesgo de desarrollar trastornos del neurodesarrollo.

También la presencia de cierta sintomatología por parte de los niños podría estar relacionada con un mayor nivel de estrés de los progenitores^35^ y, por tanto, estilos de crianza menos democráticos^30^^,^^32^. La relación entre presencia de otros niños en el hogar y los problemas de neurodesarrollos podría vincularse al trato diferencial de los progenitores hacia su descendencia^31^, hipótesis que indicaría que los problemas de neurodesarrollo son menos frecuentes cuando el trato parental es más equitativo^31^, cuya explicación más plausible reside en la teoría de la comparación social, según la cual los niños tienden a compararse con los demás, derivando su autoestima de esta comparación^36^. Por tanto, la comparación entre hermanos puede causar sentimientos de injusticia, inseguridad y ansiedad^36^^,^^37^. El estrés parental no pudo incluirse en este análisis porque lo recogimos en una visita de seguimiento posterior, pero sí se ajustó por el nivel socioeconómico, ya que se ha relacionado la posición socioeconómica con el neurodesarrollo infantil^35^. Los problemas internalizantes también podrían estar influidos por la mala relación entre hermanos^31^, aspecto que no ha podido ser valorado en esta investigación.

Los problemas del sueño son un posible mecanismo mediador en la asociación ruido-neurodesarrollo^12^^,^^16^^,^^17^. Al incluir esta variable en nuestro análisis, los estimadores apenas variaron. La literatura describe que el TDAH es el trastorno más relacionado con problemas de sueño, pero esta relación es compleja, multidireccional y multicausal^37^. Un trabajo reciente que incluye mayor muestra del Proyecto INMA, cuya fortaleza reside en que los problemas de sueño fueron medidos temporalmente antes que los relacionados con el TDAH y que, como análisis de sensibilidad, fueron excluidos los participantes con síntomas previos o concurrentes a los problemas de sueño, encontró que los problemas de sueño a los 8-9 años de edad se relacionaban con una mayor puntuación en TDAH a los 10-11 años^38^.

El presente trabajo tiene algunas limitaciones. En primer lugar, el nivel de molestia causada por el ruido se midió subjetivamente (percepción); no obstante, se considera que el ruido percibido es un buen indicador de la exposición al mismo^36^. En segundo lugar, los baremos del instrumento y puntos de corte recomendados, a pesar de aplicarse según el manual, podrían estar desfasados o no ajustarse a la población española. En tercer lugar, las molestias por ruido de otros niños fueron informados por las madres, por lo que sus factores psicológicos podrían estar sesgando la información proporcionada^37^. Además, su diseño transversal no permite detectar inferencia causal, solo asociación. Asimismo, es preciso señalar los problemas de representatividad por atrición de la nuestra; las pérdidas de seguimiento podrían estar afectando a los resultados: aquellas familias con posición social más baja abandonaron el estudio con mayor frecuencia, y son precisamente estas cuyos hijos presentaron más problemas de neurodesarrollo. Por este motivo, el impacto del ruido en el neurodesarrollo infantil podría ser incluso mayor en la población general^39^. Aunque se ha considerado el gradiente socioeconómico, no se han podido obtener indicadores urbanos o de barrio, que suelen implicar un factor de desigualdad adicional^39^. Finalmente, no ha sido posible realizar una estratificación generalizada por sexo, ya que el tamaño de la muestra no es lo suficientemente grande para realizar inferencias sólidas o generalizaciones precisas.

Como fortalezas, cabría destacar el elevado número de variables por las que se han ajustado los resultados y que este estudio, a diferencia de la mayoría, consideró diferentes fuentes de ruido e incluyó la molestia de ruido en el hogar, que está menos estudiada que en el ámbito escolar^16^.

En conclusión, los hallazgos sugieren una asociación entre la molestia por ruido y el riesgo de problemas en el neurodesarrollo de niños y niñas de 9 años de la cohorte INMA-Valencia que, además, varía según la fuente de ruido. Altos niveles de exposición a tráfico en la calle y a vecinos predijeron riesgo de TDAH, mientras que a otros niños en casa y a y actividades en la casa predijeron riesgo de problemas internalizantes y a otros niños en casa predijeron riesgo de problemas externalizantes; estos efectos no variaron al ajustar por problemas de sueño.

Se necesitan más investigaciones que apoyaran estas evidencias, utilizando como variables principales tanto la exposición al ruido evaluada objetivamente como las molestias que de este se desprenden. Estos resultados refuerzan el papel perjudicial de la contaminación acústica sobre la salud infantil, lo que indica la necesidad de actuar en pro de la prevención de trastornos del neurodesarrollo.

## Data Availability

Datos no disponibles.
